# Supplementary material for: The HIV capsid mimics karyopherin engagement of FG-nucleoporins
Source: Nature. 2024 Jan 24;626(8000):836–42. doi: 10.1038/s41586-023-06969-7 (PMC10881392; doi:10.1038/s41586-023-06969-7)
Supplement: Supplementary file 1 — Supplementary Figs. 1 and 2 and Table 1. [file 41586_2023_6969_MOESM1_ESM.pdf]

nature portfolio

https://doi.org/10.1038/s41586-023-06969-7

Supplementary information

The HIV capsid mimics karyopherin engagement of FG-nucleoporins

In the format provided by the authors and unedited

The HIV capsid mimics karyopherin engagement of FG-nucleoporins

Dickson, C.F.*, Hertel, S.*, Tuckwell, A., Li, N., Ruan, J., Al-Izzi, S.C., Ariotti, N., Sierecki, E., Gambin, Y., Morris, R.G., Towers, G.J., Böcking, T., Jacques, D.A.

*Contributed equally to this work.

Corresponding author: DA Jacques.
Email: d.jacques@unsw.edu.au

Table of Contents:

SI Figure 1 | In-gel fluorescence of cell-free expressed GFP-fusion proteins used for FFS.

SI Figure 2 | Coomassie-stained SDS-PAGE images of recombinant proteins used in this study.

SI Table 1 | Summary of studies reporting effects of Nup depletion on HIV infection and Nup:Capsid binding interactions.

Supplemental References

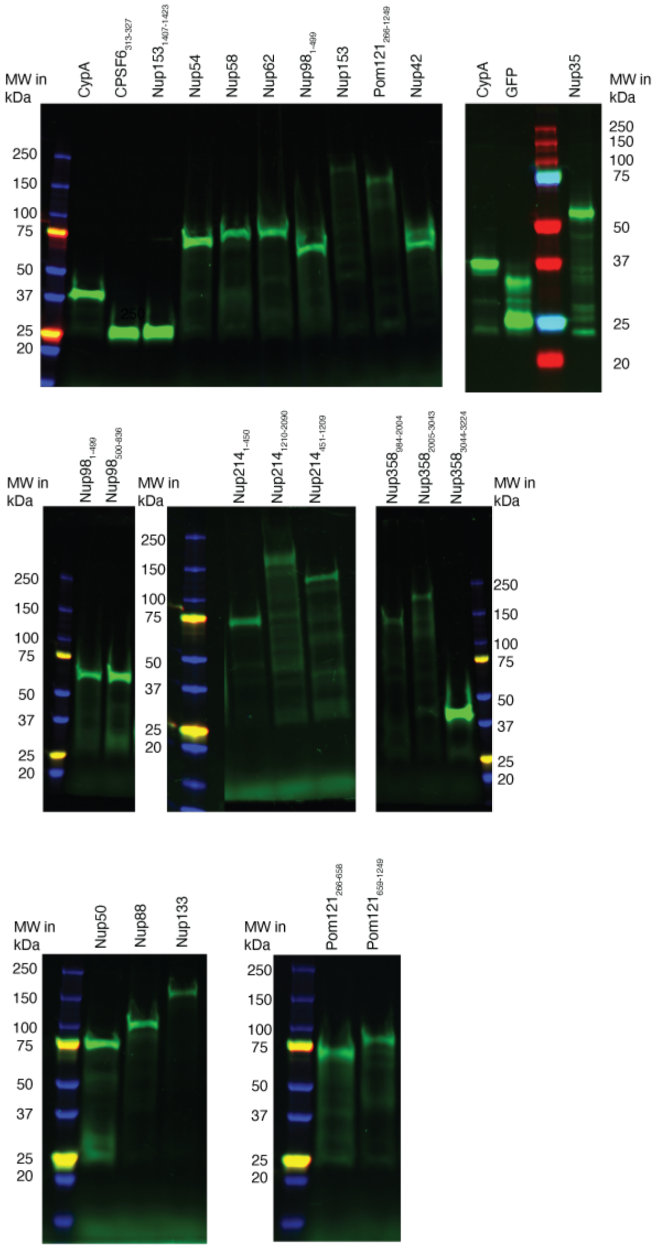

Supplementary Figure 1: In-gel fluorescence of cell-free expressed GFP-fusion proteins used for FFS.

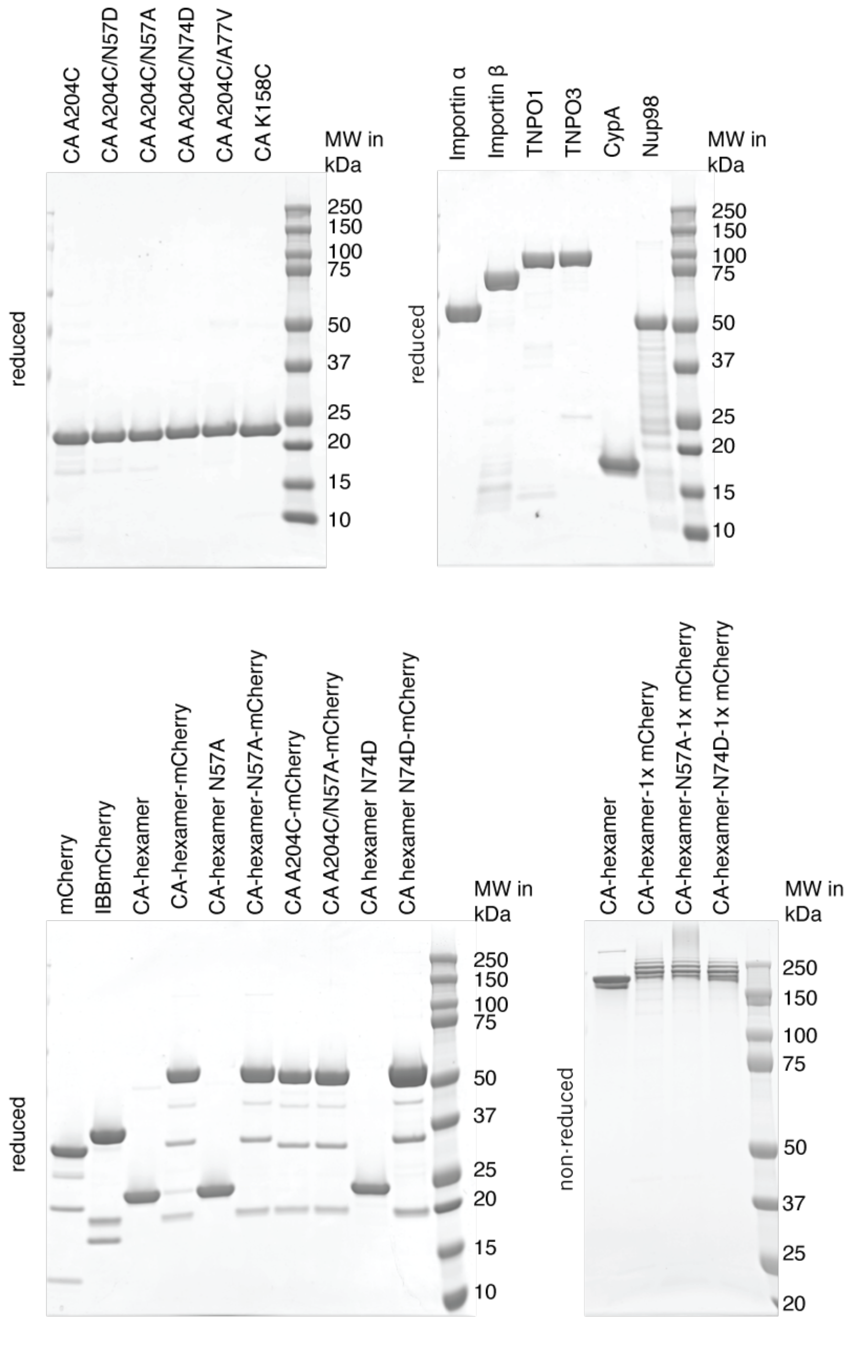

Supplementary Figure 2: Coomassie-stained SDS-PAGE images of recombinant proteins used in this study.

Supplementary Table 1: Summary of studies reporting effects of Nup depletion upon HIV infection, and identified Nup:Capsid binding interactions. **(1)** Reports of effects of Nup depletion on HIV infection compared to wild type cells. 'Y' indicates reported Nup depletion as having reduced infection levels (<2/3 WT), 'N' indicates no reported infection effects in genome wide studies or similar levels of infection (between 2/3 and 4/3 WT) for NPC wide studies, and 'N*' indicates infection was increased (>4/3 WT). **(2)** Reports of Nup:Capsid binding interactions. Sub-columns indicate interactions with various capsids: 'WT' without known FG-binding mutations, with a mutation at residue N57 that reduce binding to known FG-binders, or a N74 mutation that reduce binding to CPSF6. 'Y' and 'N' indicate whether or not binding was reported to WT and 'Y-' or 'Y+' indicates whether a mutation increased or decreased binding. Blank cells indicate that Nup:Capsid interaction experiment for these conditions was not performed. ^ Saito *et al.* did not detect HIV cores binding to Pom12C but found a negative impact on HIV infection with overexpression of Pom121C$_{612-987}$ in multiple cell lines.

**1. HIV Infection Impaired by Nup Depletion**			**FG-Repeat Nups**										**Non-FG Nups**		
**First author**	**Year**	**Experimental Method**	**Nup42**	**Nup50**	**Nup54**	**Nup58**	**Nup62**	**Nup98**	**Pom121**	**Nup153**	**Nup214**	**Nup358**	**Nup35**	**Nup88**	**Nup133**
Brass *et al.*[34]	2008	Genome wide knock down in Hela cells	N	N	N	N	N	N	N	Y	N	N	N	N	Y
König *et al.*[35]	2008	Genome wide knock down in 293T cells	N	Y	N	N	Y	Y	N	Y	Y	N	N	N	N
Zhou *et al*[62].	2008	Genome wide knock down in Hela cells	N	N	N	N	N	N	N	N	N	N	N	N	N
Yeung *et al*[63].	2009	Genome wide knock down in Jurkat cells	N	N	N	N	N	Y	N	N	N	N	N	N	N
Ebina *et al*[64].	2004	Targeted knock down in 293T cells						Y							
Lee *et al.*[21]	2010	Targeted knock down in Hela cells								Y					N
Matreyek *et al*[65].	2011	Targeted knock down in Hela cells								Y					
Di Nunzio *et al.*[33]	2012	Targeted knock down in Hela cells						Y		Y	Y	Y			
Ao *et al.*[66]	2012	Targeted knock down in C8166/macrophages cells					Y								
Matreyek *et al.*[15]	2013	Targeted knock down in HOS cells								Y					
Di Nunzio *et al.*[67]	2013	Targeted knock down in Jurkat cells						Y		Y					
Dharan *et al.*[68]	2016	Targeted knock down in Hela cells										Y			
Guo *et al.*[69]	2018	Targeted knock down in 293T/C8166 cells							Y						
Buffone *et al*[20].	2018	Targeted knock down in Hela cells					Y			Y	Y	Y			
Kane *et al.*[19]	2018	NPC wide knock down in Hela cells		N	N		N	Y		Y	Y	Y	N	N	N
Xue *et al.*[36]	2023	NPC wide knock down in Hela cells		Y	N*	N*	N*	Y	Y	Y	N*	Y	Y	N*	Y

**2. Nup:Capsid Binding Interactions**			**FG-Repeat Nups**																												**Non-FG Nups**								
**First author**	**Year**	**Experimental Method**	**Nup42**			**Nup50**			**Nup54**			**Nup58**			**Nup62**			**Nup98**			**Pom121**			**Nup153**			**Nup214**			**Nup358**			**Nup35**			**Nup88**			**Nup133**		
			WT	N57	N74	WT	N57	N74	WT	N57	N74	WT	N57	N74	WT	N57	N74	WT	N57	N74	WT	N57	N74	WT	N57	N74	WT	N57	N74	WT	N57	N74	WT	N57	N74	WT	N57	N74	WT	N57	N74
Schaller *et al*[6].	2011	Isothermal calorimetry of Nup358CypA domain and CA NTD																												Y											
Di Nunzio *et al*[33].	2012	Pull-downs from 293T cell lysate, *in vitro* HIV1 CA-NC complex																												Y											
Di Nunzio *et al.*[67]	2013	Pull-downs from 293T cell lysate, *in vitro* HIV1 CA-NC complex																Y		Y-				Y		Y															
Matreyek *et al.*[15]	2013	Pulldown from 293T cell lysate and recombinant Nup153C, *in vitro* HIV1 CA-NC complex																						Y	Y-	Y+															
Bhattacharya *et al.*[70]	2014	Fluorescence anisotropy of fNup153 peptide and HIV-1 CA hexamers																						Y																	
Saito *et al.*[37]	2017	Immunoprecipitation of HIV Cores with Pom121C																			N^																				
Buffone *et al.*[20]	2018	Pull-downs from 293T cell lysate, *in vitro* HIV1 CA-NC complex													Y	Y-	Y+	Y	Y-					Y	Y-		Y	Y-	Y+	Y											
Kane *et al.*[19]	2018	Pull-downs from HeLa cell lysate, *in vitro* CA tubes													Y									Y						Y						Y			N		
Shen *et al.*[71]	2023	Co-pelleting of Recombinant Nup with *in vitro* CA tubes													Y									Y						Y											
Xue *et al.*[36]	2023	Pull-downs from 293T cell lysate, *in vitro* HIV1 CA complex																			Y		Y										Y		Y						
**This Study**	**2023**	**FFS of eukaryotic cell-free Nup expression, *in vitro* A204C stabilised CLPs**	**Y**	**Y-**	**Y+**	**N**			**N**			**Y**	**Y-**	**Y+**	**Y**	**Y-**	**Y+**	**Y**	**Y-**	**Y+**	**Y**	**Y-**	**Y+**	**Y**	**Y-**	**Y+**	**Y**	**Y-**	**Y+**	**Y**			**N**			**N**			**N**		

Supplementary References

62. Zhou, H. *et al.* Genome-scale RNAi screen for host factors required for HIV replication. *Cell Host Microbe* **4**, 495–504 (2008).
63. Yeung, M. L., Houzet, L., Yedavalli, V. S. R. K. & Jeang, K. T. A genome-wide short hairpin RNA screening of Jurkat T-cells for human proteins contributing to productive HIV-1 replication. *Journal of Biological Chemistry* **284**, 19463–19473 (2009).
64. Ebina, H., Aoki, J., Hatta, S., Yoshida, T. & Koyanagi, Y. Role of Nup98 in nuclear entry of human immunodeficiency virus type 1 cDNA. *Microbes Infect* **6**, 715–724 (2004).
65. Matreyek, K. A. & Engelman, A. The requirement for nucleoporin NUP153 during human immunodeficiency virus type 1 infection is determined by the viral capsid. *J Virol* **85**, 7818–7827 (2011).
66. Ao, Z. *et al.* Contribution of host nucleoporin 62 in HIV-1 integrase chromatin association and viral DNA integration. *Journal of Biological Chemistry* **287**, 10544–10555 (2012).
67. Di Nunzio, F. *et al.* Nup153 and Nup98 bind the HIV-1 core and contribute to the early steps of HIV-1 replication. *Virology* **440**, 8–18 (2013).
68. Dharan, A. *et al.* KIF5B and Nup358 cooperatively mediate the nuclear import of HIV-1 during infection. *PLoS Pathog* **12**, e1005700 (2016).
69. Guo, J. *et al.* The transmembrane nucleoporin Pom121 ensures efficient HIV-1 pre-integration complex nuclear import. *Virology* **521**, 169–174 (2018).
70. Bhattacharya, A. *et al.* Structural basis of HIV-1 capsid recognition by PF74 and CPSF6. *Proceedings of the National Academy of Sciences* **111**, 18625–18630 (2014).
71. Shen, Q. *et al.* Modeling HIV-1 nuclear entry with nucleoporin-gated DNA-origami channels. *Nat Struct Mol Biol* **30**, 425–435 (2023).
