# Supplementary material for: The HIV capsid mimics karyopherin engagement of FG-nucleoporins
Source: Nature. 2024 Jan 24;626(8000):836–42. doi: 10.1038/s41586-023-06969-7 (PMC10881392; doi:10.1038/s41586-023-06969-7)
Supplement: Supplementary file 2 — Reporting Summary [file 41586_2023_6969_MOESM2_ESM.pdf]

nature portfolio

Corresponding author(s): David Jacques

Last updated by author(s): Oct 17, 2023

Reporting Summary

Nature Portfolio wishes to improve the reproducibility of the work that we publish. This form provides structure for consistency and transparency in reporting. For further information on Nature Portfolio policies, see our [Editorial Policies](#) and the [Editorial Policy Checklist](#).

Statistics

For all statistical analyses, confirm that the following items are present in the figure legend, table legend, main text, or Methods section.

n/a	Confirmed	
☐	☒	The exact sample size (*n*) for each experimental group/condition, given as a discrete number and unit of measurement
☐	☒	A statement on whether measurements were taken from distinct samples or whether the same sample was measured repeatedly
☐	☒	The statistical test(s) used AND whether they are one- or two-sided<br>*Only common tests should be described solely by name; describe more complex techniques in the Methods section.*
☒	☐	A description of all covariates tested
☒	☐	A description of any assumptions or corrections, such as tests of normality and adjustment for multiple comparisons
☐	☒	A full description of the statistical parameters including central tendency (e.g. means) or other basic estimates (e.g. regression coefficient) AND variation (e.g. standard deviation) or associated estimates of uncertainty (e.g. confidence intervals)
☐	☒	For null hypothesis testing, the test statistic (e.g. *F*, *t*, *r*) with confidence intervals, effect sizes, degrees of freedom and *P* value noted<br>*Give P values as exact values whenever suitable.*
☒	☐	For Bayesian analysis, information on the choice of priors and Markov chain Monte Carlo settings
☒	☐	For hierarchical and complex designs, identification of the appropriate level for tests and full reporting of outcomes
☒	☐	Estimates of effect sizes (e.g. Cohen's *d*, Pearson's *r*), indicating how they were calculated

Our web collection on [statistics for biologists](#) contains articles on many of the points above.

Software and code

Policy information about [availability of computer code](#)

Data collection	Fluorescence Fluctuation Spectroscopy data were collected using LabVIEW 14.0f1<br>Confocal Microscopy Data were collected using Zen 2.1 from Zeiss<br>CryoEM tilt series were collected using Tomography (Thermo Fisher Scientific, version 5.12.0.4776REL)
Data analysis	Fluorescence Fluctuation Spectroscopy data were analysed using TRISTAN v0_2 (Two Reagents Incident Spectroscopic Analysis, freely available on https://github.com/lilbutsa/Tristan<br>Averaged radial intensity profiles were obtained using the plugin Radial Profile in ImageJ Version 2.9.0/1.53t<br>Three-dimensional reconstructions from cryoEM tilt seris were generated with IMOD 4.11.19<br>Matlab Version R2019b and GraphPad Prism 9.4.1 were used for statistical analysis and data presentation

For manuscripts utilizing custom algorithms or software that are central to the research but not yet described in published literature, software must be made available to editors and reviewers. We strongly encourage code deposition in a community repository (e.g. GitHub). See the Nature Portfolio [guidelines for submitting code & software](#) for further information.

Data

Policy information about availability of data

All manuscripts must include a data availability statement. This statement should provide the following information, where applicable:

- Accession codes, unique identifiers, or web links for publicly available datasets
- A description of any restrictions on data availability
- For clinical datasets or third party data, please ensure that the statement adheres to our policy

The experimental data that support the findings of this study are available at Dryad with the identifier: https://doi.org/10.5061/dryad.b2rbnzsm0

Research involving human participants, their data, or biological material

Policy information about studies with human participants or human data. See also policy information about sex, gender (identity/presentation), and sexual orientation and race, ethnicity and racism.

Reporting on sex and gender	N/A
Reporting on race, ethnicity, or other socially relevant groupings	N/A
Population characteristics	N/A
Recruitment	N/A
Ethics oversight	N/A

Note that full information on the approval of the study protocol must also be provided in the manuscript.

Field-specific reporting

Please select the one below that is the best fit for your research. If you are not sure, read the appropriate sections before making your selection.

☒ Life sciences ☐ Behavioural & social sciences ☐ Ecological, evolutionary & environmental sciences

For a reference copy of the document with all sections, see nature.com/documents/nr-reporting-summary-flat.pdf

Life sciences study design

All studies must disclose on these points even when the disclosure is negative.

Sample size	This is a biophysical study on purified chemicals and did not involve predetermined sample sizes of individual participants or animals. Fluorescence Fluctuation Spectroscopy is a single molecule technique in which thousands to millions of individual events are recorded in a single experiment, with experiments repeated at least twice giving statistical power. Confocal microscopy observed tens to hundreds of individual nucleoporin condensates over multiple fields of view per experiment. Null hypothesis testing gave p values < 10^-10 demonstrating statistical power. Tens of Cryo-electron tomography tilt series were collected per sample to illustrate nanoscale capsid behavior, with the sample size chosen to optimise use of electron microscope time.
Data exclusions	For Fluorescence Fluctuations Spectroscopy data were excluded if systematic baseline variation occurred (indicating instument drift and/or misalignment).<br>For confocal microscopy analysis, condensates too small to appropriately analyse were not considered, intensity values which significantly differed from the average value were discarded
Replication	Results were replicated at least twice.
Randomization	Randomization was not relevant to the study of capsids interacting with nucleoporins as this is a biophysical study on purified chemicals, and does not involve individual human or animals that require randomization following assessment of study eligibility.
Blinding	Randomization was not relevant to the study of capsids interacting with nucleoporins as this is a biophysical study on purified chemicals, and there are no individual participants that can be influenced by information regarding the experiment.

Reporting for specific materials, systems and methods

We require information from authors about some types of materials, experimental systems and methods used in many studies. Here, indicate whether each material, system or method listed is relevant to your study. If you are not sure if a list item applies to your research, read the appropriate section before selecting a response.

Materials & experimental systems

n/a	Involved in the study
☒	☐ Antibodies
☐	☒ Eukaryotic cell lines
☒	☐ Palaeontology and archaeology
☒	☐ Animals and other organisms
☒	☐ Clinical data
☒	☐ Dual use research of concern
☒	☐ Plants

Methods

n/a	Involved in the study
☒	☐ ChIP-seq
☒	☐ Flow cytometry
☒	☐ MRI-based neuroimaging

Eukaryotic cell lines

Policy information about cell lines and Sex and Gender in Research

Cell line source(s)	HEK293T cells were obtained from ATCC
Authentication	Live cells were not used in this study and were only used as a source of specific cDNAs, which were verified by Sanger sequencing
Mycoplasma contamination	None
Commonly misidentified lines (See ICLAC register)	N/A

Plants

Seed stocks	N/A
Novel plant genotypes	N/A
Authentication	N/A
